# Editorial: Green Synthesis of Heterocycles

**DOI:** 10.3389/fchem.2020.00074

**Published:** 2020-02-12

**Authors:** Fabio Aricò

**Affiliations:** Department of Environmental Sciences, Informatics and Statistics, Ca' Foscari University, Venice, Italy

**Keywords:** green chemistry, heterocycles, cyclic compound, sustainable, green principles

## Introduction

Over the last two centuries, new approaches to the synthesis of heterocycles have had an enormous impact on both organic and inorganic chemistry. Natural products, renewable resources, agrochemicals, pharmaceuticals, and macromolecules (polymers and macrocycles) often feature heterocyclic substructures. Approaches to the synthesis of these compounds have been evolving constantly from classical condensation procedures to click reactions and new multicomponent domino procedures. Furthermore, the development of new approaches to heterocycle synthesis has been a major research interest for green and sustainable chemists.

In this perspective, the Research Topic “Green Synthesis of Heterocycles” encompasses a collection of research and review articles focusing on heterocyclic compounds synthetized according to Green Chemistry principles. The focal point was to build on efficient catalytic methodologies aiming at high process performances by means of non-toxic/green and biodegradable chemicals. Industrial applications, process developments, and future perspectives of the so-synthetized heterocyclic compounds have also been addressed.

This collection of articles features two reviews and six original research papers.

The first review focuses on the synthesis of heterocycles via 1,3-dipolar cycloaddition employing Green approaches (Martina et al.). The authors report on the preparation of numerous cyclic structures, including pyrrolizidines pyrrolo[2,3-a]pyrrolizidino derivatives, isoxazolidines, pyrazoles, pyrrolidines and ispirooxindolopyrrolidines, diazaheptacyclic rings, etc. The use of green solvents, i.e., ionic liquids, fluorinated solvents, and water is discussed. Improvements over commonly used organic solvents are highlighted and, in specific cases, the enhancement in regio- and stereo-selectivity addressed. Besides, catalyst-free reactions and alternative reaction conditions—such as microwave irradiation and activation by light exposure—are also reported for several substrates.

The second review is centered on the use of dialkyl carbonates (DACs) as green reagent and solvent molecules for the synthesis of several heterocycles (Tundo et al.). The preparation of tetrahydrofuran systems, pyrrolidines, indolines, isoindoline, and 1,4-dioxanes was generally conducted in the presence of a base and dimethyl carbonate (DMC). Interestingly, a new class of compounds, namely, mustard carbonates was explored for the synthesis of piperidines. In the case of bio-based platform chemical 5-hydroxymethylfurfural (HMF), DMC was employed as an efficient extracting solvent. In all the reported cyclizations, DACs acted as reaction media and as a sacrificial molecule, mimicking the typical reactivity of chlorine compounds but without the intrinsic toxicity of halogen-based molecules.

Some of the major issues in heterocycle synthesis are the recycling of solvent/catalyst systems and avoiding purification and, consequently, waste production. In this view, one of the original research papers in this collection reports on isoxazole and 1,2,3-triazole preparation by developing efficient protocols that feature recycling of both solvent and catalyst. Within this scope, Polarclean, an alternative to a classical polar solvent, is shown to be an efficient medium in dipolar cycloaddition reactions and intramolecular C–H activation to synthetize isoxazoles and hetero-fused triazoles, respectively (Ferlin et al.). Both cyclization reactions were conducted in one step, in an atom economical fashion, minimizing waste generation. Isolation of the product was achieved via re-crystallization without the need for further purification. Most importantly, the solvent/catalyst system could be effectively reused.

The preparation of 1,2,3-triazoles, and specifically 1,4-disubstituted-1,2,3-triazoles, is also addressed in the research article by Aflak et al.. In this work, triazoles were prepared selectively via click chemistry by copper on carbon-catalyzed [3+2] cycloaddition reactions of azides and alkynes in water. Catalytic systems employed for the reaction were based on copper(I) catalysts heterogenized onto commercially activated carbon materials (Cu-CC) and on carbon material produced from vegetable biomass using Argan nut shells (Cu-CANS). In the reaction conditions found to be most optimal, a series of azides and alkynes were reacted in water in the presence of the new catalytic system (0.5 mol %) at room temperature. The reported heterogeneous copper on carbon catalysts were recovered and reused for up to 10 catalytic runs with minor activity loss.

A topic that is attracting growing attention is the preparation of bio-based platform chemicals via biomass valorization. One of the published research papers focuses on the catalytic conversion of levulinic acid into N-heterocycles (Rodríguez-Padrón et al.). The catalytic system employed was achieved from graphitic carbon nitride (g-C_3_N_4_) functionalized with a low platinum loading (g-C_3_N_4_/Pt). The catalytic performance of the so-prepared material was investigated in the continuous-flow transformation of levulinic acid to N-heterocycles. The reaction conditions (temperature, pressure, and concentration of starting compound) were optimized. The catalytic system displayed high selectivity for the preparation of 1-ethyl-5-methylpyrrolidin-2-one and very good stability after 3 h of reaction.

Oxazinanones (six-membered cyclic urethanes) are interesting heterocycles that are key structural units in bioactive natural products and pharmaceuticals. The research study reported by Gallo et al. focuses on the synthesis of 1,3-oxazinane-2,5-diones via acid catalyzed intramolecular cyclization of N-Cbz-protected diazoketones derived from α-amino acids. The reaction was carried out in metal-free conditions, being promoted by a silica-supported catalyst and with methanol as the solvent. In a typical reaction, diazo carbonyl reagent was mixed with the catalyst at room temperature, and a good yield of the products was achieved (90%); no further purifications were required.

Flash vacuum pyrolysis (FVP) has recently been reported as a viable technique for the synthesis of several heterocycles. In this view, one of the research articles focuses on a gas-phase reaction of 1,3-dithiolane-2-thione over molybdenum trioxide supported on pumice stone that resulted in its conversion into 1,3-dithiolan-2-one (Aitken et al.). The reaction was carried out by physical mixing of MoO_3_ and pumice or by solution impregnation, resulting, under the reaction conditions found to be most optimal, in an isolated yield of up to 67%. At the end of the reaction, besides the wanted 1,3-dithiolan-2-one, partially sulfurized MoO_3_ is recovered; the latter is regenerated to the active catalyst on exposure to air.

The development of sustainable synthetic methods for the construction of complicated cyclic target molecules with minimum environmental impacts is a challenging aim for academia and industry. In this view, the article by Jang and co-workers focuses on cyclobuta[a]naphthalen-4-ols construction employing a bicyclization reaction of yne-allenones with indoles (Li et al.). This reaction was performed using commercially available FeCl_3_ as the catalyst and EtOH as a benign solvent. The reaction proceeded via a [2+2] cycloaddition/1,6-conjugate addition cascade, which led to a good yield of the desired product (up to 90%). This protocol has the advantages of a broad scope of substrates, good tolerance of functional group, and high atom utilization together with mild reaction conditions.

The present article collection illustrates some of the recent advances in heterocycle preparation that employ more sustainable synthetic procedures. This topic is particularly important for drug development since the pharmaceuticals industry is environmentally very problematic. As a result, investigations on greener approaches to heterocyclic compounds are gaining ever-growing attention in the scientific community. The most relevant future directions to achieve this goal are evidently depicted in the above-summarized articles, i.e., employing multicomponent reactions, using green/renewable-based solvents, and heterogeneous catalysis, better if in combination with alternative reaction conditions. Besides, waste consumption should be reduced, and any purification avoided.

## Author Contributions

The author confirms being the sole contributor of this work and has approved it for publication.

### Conflict of Interest

The author declares that the research was conducted in the absence of any commercial or financial relationships that could be construed as a potential conflict of interest.

